# Droplet digital PCR assay for precise determination of FRS2 gene copy number in bladder cancer

**DOI:** 10.1186/s12885-025-14611-0

**Published:** 2025-07-24

**Authors:** Jinqian Li, Jiaqi Liang, Yinyan Xu, Wei Tan, Guanzheng Chen, Tong Ou

**Affiliations:** 1 Medical Laboratory, Shenzhen Luohu People’s Hospital, Shenzhen, Guangdong 518000 China; 2 NHC Key Laboratory of Tropical Disease Control, School of Tropical Medicine, Hainan Medical University, Haikou, Hainan 571199 China; 3 Luohu Clinical College, Shantou University Medical College, Shenzhen, Guangdong 518000 China; 4 Medical Laboratory Center, Shenzhen Luohu Hospital Group, Shenzhen, Guangdong 518000 China

**Keywords:** Bladder cancer, Gene copy number, Formalin-fixed paraffin-embedded (FFPE), FRS2, Droplet digital PCR (ddPCR)

## Abstract

**Objective:**

To establish and validate a droplet digital PCR (ddPCR) assay for quantifying FRS2 gene copy number in formalin-fixed paraffin-embedded (FFPE) bladder cancer tissue samples, and to evaluate its analytical performance.

**Methods:**

The ddPCR assay was developed using FRS2 as the target gene and RPP30 as the reference gene. Artificial plasmids, genomic DNA from urinary sediment of healthy individuals and cell lines were used as templates to assess the assay’s precision, minimum reliable input DNA, and linearity. Fluorescence in situ hybridization (FISH) was employed to validate the accuracy of the ddPCR results.

**Results:**

One-dimensional fluorescence amplitude plots showed clear separation between positive and negative droplets for both FRS2 and RPP30. Duplex detection of FRS2 and RPP30 within the same reaction showed no interference between primers or probes. The assay exhibited excellent repeatability and precision, with intra-assay coefficient of variation (CV)% of 2.58% and 3.75%, and inter-assay CV% of 2.68% and 3.79%, across 20 ng and 2 ng input levels, respectively. The minimum reliable input DNA amount was determined to be 2 ng, and a strong linear relationship was observed (R^2^ >0.99). Compared to FISH, the ddPCR assay showed 100% sensitivity, 100% specificity, and a kappa value of 1.

**Conclusion:**

The developed ddPCR assay enables accurate and reliable quantification of FRS2 copy number in FFPE samples, offering a promising tool for auxiliary diagnosis and prognostic assessment in bladder cancer.

**Supplementary Information:**

The online version contains supplementary material available at 10.1186/s12885-025-14611-0.

## Introduction

Bladder cancer (BCa) is one of the most common malignancies of the urinary system, characterized by a high incidence, frequent genomic alterations, and a strong tendency for recurrence [1–3]. Clinically, BCa is stratified into non-muscle-invasive (NMIBC) and muscle-invasive (MIBC) subtypes based on tumor invasiveness [4, 5].

The development and progression of BCa are driven by a complex array of genomic alterations. In recent years, substantial advances have been made in elucidating the complex and heterogeneous molecular landscape of bladder cancer [6, 7]. Among these alterations, gene-level copy number alterations (CNAs) have emerged as key drivers of dysregulated gene expression and aberrant activation of tumor-related signaling pathways [8]. CNAs have been identified in over 20% of NMIBC and up to 30% of MIBC cases [9], indicating their significance in disease progression. Accordingly, the integration of molecular CNAs profiling into clinical practice holds promise for improving risk stratification and facilitating the implementation of personalized therapeutic strategies [9].

Fibroblast growth factor receptor substrate 2 (FRS2) is a key adaptor protein in the FGFR signaling cascade, mediating downstream RAS-MAPK and PI3K-AKT activation, and promoting tumor proliferation and survival [10, 11]. Amplification of FRS2 has been frequently observed across various malignancies, including uterine sarcoma, high-grade liposarcoma, osteosarcoma, as well as breast and ovarian cancers. Such amplification is often associated with increased tumor aggressiveness and poor clinical outcomes [10, 12–17]. Data from The Cancer Genome Atlas (TCGA) indicate that FRS2 amplification occurs in a substantial subset of patients, suggesting its potential role as a driver genetic alteration [18]. Our previous whole-genome sequencing study further demonstrated that FRS2 copy number can increase by 3- to 25-fold in bladder cancer tissues, and this amplification correlates with elevated microvessel density and adverse prognosis [19]. Collectively, these findings underscore the potential of FRS2 as a biomarker for risk stratification and as a promising target for anti-angiogenic therapy in BCa. Accurate quantification of FRS2 copy number may facilitate early diagnosis, disease monitoring, and individualized therapy.

Droplet digital PCR (ddPCR) is an emerging method for copy number determination that is gaining popularity. Unlike conventional qPCR, ddPCR partitions the PCR reaction mixture into thousands of oil-wrapped nano-sized droplets, each serving as an independent amplification microreactor. After end-point amplification, droplets are classified as positive or negative based on fluorescence signal, and target concentrations are calculated using poisson statistics without the need for standard curves or Ct values [20, 21]. This unique partitioning strategy significantly enhances quantification accuracy, sensitivity, and reproducibility. ddPCR has shown robust performance in diverse applications, including the detection of mitochondrial DNA [22], tumor-derived cell-free DNA [23], and viral genomes [24, 25].

In this study, we developed a ddPCR assay to quantify FRS2 copy number and validated its analytical performance. This platform offers a sensitive and reliable approach for detecting FRS2 amplification in bladder cancer.

## Materials and methods

### Clinical samples and DNA templates

Seventeen FFPE bladder cancer tissue samples were obtained from patients diagnosed at Shenzhen Luohu People’s Hospital, as well as 18 urine samples (≥ 10 mL) collected from healthy individuals undergoing routine physical examinations. Genomic DNA was extracted from cancer cell lines and urinary sediments using the TIANamp Genomic DNA Kit (DP304), and from FFPE tissues using the FFPE DNA Kit (DP330), following the manufacturer’s protocols. DNA concentration and purity were assessed using a NanoDrop OneC spectrophotometer (Thermo Fisher Scientific, Inc.), and all DNA samples were stored at − 80 °C until further analysis. This study was approved by the Research Ethics Committee of Shenzhen Luohu People’s Hospital, and written informed consent was obtained from all participants.

### Primers and probes

Primers and probes targeting the FRS2 gene were designed using Primer 5.0 software, while sequences for the reference gene RPP30 were adopted from established literature [26]. The primer and probe sequences used in this study are listed in Table 1. All oligonucleotides were synthesized by Guangzhou Ruibo Biotech Co., Ltd.

**Table 1:** Primer and probe sequences for FRS2 and RPP30

Target	Sequence (5’–3’)
FRS2 forward primer	5’-GCCTACAACTCCCCTTCCAC-3’
FRS2 reverse primer	5’-TCATCTCGTGGCAGTGCTTT-3’
FRS2 probe	5’-FAM-TTGACATAGCAGCAGTTCTCTCGA-BHQ-3’
RPP30 forward primer	5’-AGATTTGGACCTGCGAGCG-3’
RPP30 reverse primer	5’-GAGCGGCTGTCTCCACAAGT-3’
RPP30 probe	5’-ROX-CTGACCTGAAGGCTCT-BHQ1-3’

### Droplet digital polymerase chain reaction

ddPCR was performed using DropXpert S6 system performs according to manufacturer’s instructions [27, 28]. In brief, the PCR reaction mixture (Table 2) is pipetted into C4 chips, which are then sealed using Pressure-permeable connection caps. The chips are then placed onto the chip holder of the Droplet digital PCR system (DropXpert S6). After loading, the program will run the whole process. PCR cycling conditions: 50 °C for 10 min, 95 °C for 10 min, followed by 40 cycles of 95 °C for 10 s and 58 °C for 45 s. After cycling, the instrument will automatically detect and analyze the amplification products. The FRS2 amplification ratio was defined as: Ratio = FRS2 copy number/RPP30 copy number.

**Table 2:** ddPCR reaction system

Component	Volume (µL)
2× Aplµs™ Digital PCR Mix	10µL
DNA template	2µL
FRS2 forward primer (10 µM)	1µL
FRS2 reverse primer (10 µM)	1µL
FRS2 probe (10 µM)	0.8µL
RPP30 forward primer (10 µM)	1µL
RPP30 reverse primer (10 µM)	1µL
RPP30 probe (10 µM)	0.8µL
DEPC-treated water	2.4µL

### Minimum reliable input and precision

To assess the minimum reliable input of the ddPCR assay, genomic DNA extracted from urine sediment of healthy individuals was used as the template. Total input DNA amounts of 20 ng, 2 ng, and 0.2 ng were tested, each in triplicate. The coefficient of variation (CV) was calculated for each DNA concentration. The lowest DNA input that resulted in a CV% less than 5% was defined as the minimum reliable input.

For precision assessment, 20 ng and 2 ng of healthy urine-derived genomic DNA were used as templates. Each sample was measured in triplicate per day for five consecutive days. Intra-assay CV% and Inter-assay CV% were calculated to evaluate the precision of the assay.

### Fluorescence in situ hybridization (FISH)

A custom-designed dual-probe FISH assay was developed, targeting the FRS2 gene and the centromeric region of chromosome 12 (CEP12). The FRS2 probe was labeled with a red fluorophore and the CEP12 probe with a green fluorophore. In brief, formalin-fixed, paraffin-embedded bladder cancer tissue sections were deparaffinized, rehydrated, and subjected to heat-induced epitope retrieval and DNA denaturation, followed by overnight hybridization with the probe mix at 37 °C. After stringent washes and DAPI counterstaining, fluorescence signals were analyzed under a fluorescence microscope. For each sample, signals from 25 randomly selected tumor nuclei were counted. A case was considered FISH-positive if the FRS2/CEP12 ratio was ≥ 2.0 and the average FRS2 copy number per cell was ≥ 4.0.

### FRS2 mutation characterization

To investigate the mutational landscape of FRS2 across various cancer types, we utilized the cBioPortal for Cancer Genomics platform (https://www.cbioportal.org, accessed on April 5, 2025). We first selected the TCGA Pan-Cancer Atlas study cohort as the data source. Subsequently, the gene symbol “FRS2” was entered into the “Query” module to retrieve comprehensive information on the genomic alterations of FRS2. The “OncoPrint”, “Cancer Types Summary” and “Comparisons/Survival” modules were used to examine the frequency, types, and the association between FRS2 mutations and patient survival outcomes in multiple cancer cohorts.

### Statistical analysis

Statistical analyses were conducted in Excel, and plotting was performed using GraphPad Prism 9.0 for Windows (GraphPad Software, San Diego, CA, USA). The CV was calculated as the ratio of the standard deviation to the mean (CV = SD/Mean × 100%). The intra-assay and inter-assay coefficients of variation (CV%) were calculated in accordance with the CLSI EP05-A3 guideline.

## Result

### The characteristics of FRS2 mutations in the TCGA pan-cancer cohort

**Fig. 1:**
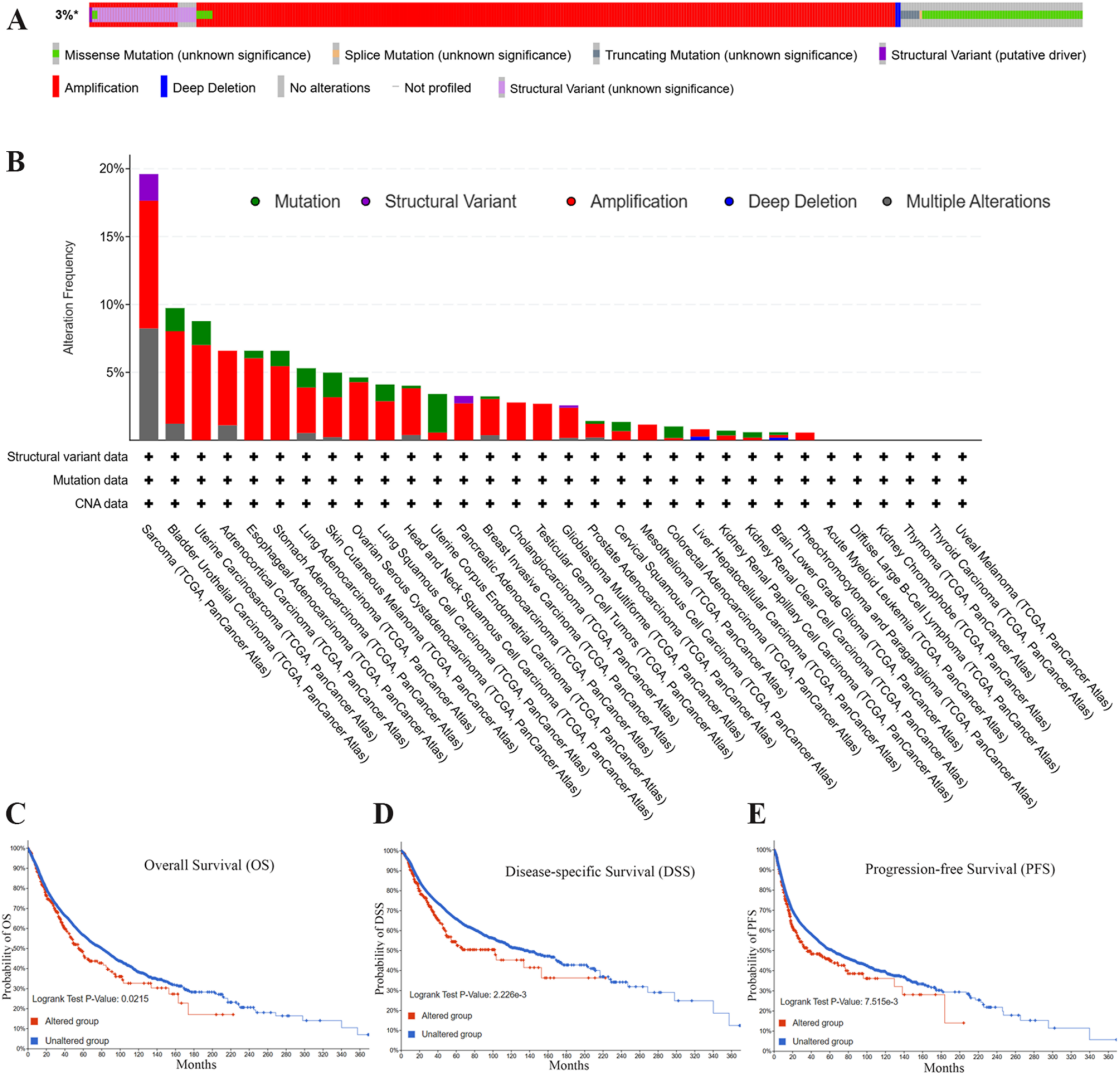
FRS2 mutations in tumors. **A** OncoPrint visual summary of alterations in a query of FRS2 from cBioPortal. **B** Frequency and type of mutations in the FRS2 gene change in pan-cancer. **C**-**E** The association between FRS2 genetic alterations and clinical survival prognosis. **C** Overall Survival, **D** Disease-specific Survival, **E** Progression-free Survival

We analyzed the mutational landscape of the FRS2 gene across multiple cancer types using the cBioPortal platform. As shown in Fig. 1A, FRS2 alterations were identified in 329 of 10,967 tumor samples, representing approximately 3% of all cases. These alterations, predominantly gene amplifications, appeared in 26 cancer types as illustrated in Fig. 1B. The highest alteration frequencies were observed in sarcoma (SARC), bladder urothelial carcinoma (BLCA), and uterine carcinosarcoma (UCS), whereas no alterations were found in acute myeloid leukemia (LAML), diffuse large B-cell lymphoma (DLBC), kidney chromophobe (KICH), thymoma (THYM), thyroid carcinoma (THCA), or uveal melanoma (UVM).

Survival analysis further demonstrated that FRS2 alterations were significantly correlated with poorer clinical outcomes, including overall survival (OS, *p* < 0.05), disease-specific survival (DSS, *p* < 0.01), and progression-free survival (PFS, *p* < 0.01) (Fig. 1C–E), while no significant correlation was observed with disease-free survival (DFS, *p* = 0.35) (data not shown).

### Development of a ddPCR assay for FRS2 copy number detection

**Fig. 2:**
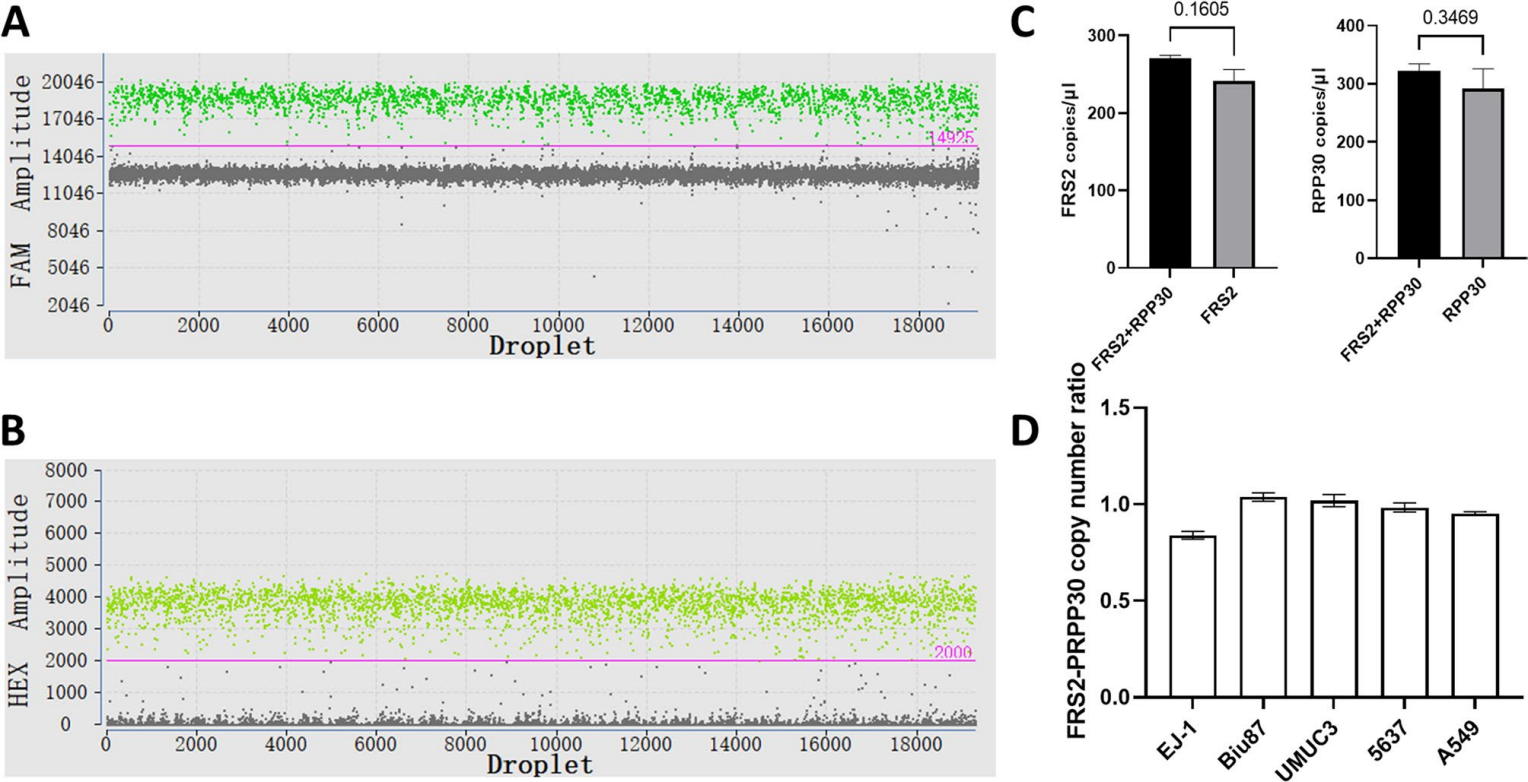
Droplet plots of the ddPCR assay. **A**, **B** One-dimensional amplitude plot. **A** FRS2-positive and -negative droplets are shown as dark green and gray dots, respectively; **B** RPP30-positive and -negative droplets are shown as light green and gray dots, respectively; **C** Comparison of singleplex and duplex assays for FRS2 and RPP30 detection. Black bars represent duplex reactions; gray bars represent singleplex reactions. Copy number results are presented as mean ± SD from three independent replicates. Statistical differences were assessed using Student’s t-test; **D** Validation of RPP30 as a reference gene using cancer cell lines without FRS2 amplification. Data are shown as mean ± SD from three independent replicates

Accurate assessment of gene amplification by ddPCR requires a reference gene with a stable copy number for normalization. According to previously published results from GISTIC analysis of 10,844 tumor samples, RPP30 shows no significant copy number alterations and has therefore been widely adopted as a reliable reference gene in copy number studies [26, 29, 30]. In this study, RPP30 was validated as an internal control for a duplex ddPCR assay targeting FRS2. Clear separation between positive and negative droplets was observed in both detection channels (Fig. 2A, B). To assess potential interference, we compared the copy numbers of FRS2 and RPP30 in both singleplex and duplex assays. The results showed no significant difference between the two setups (*p* > 0.05) (Fig. 2C), indicating that combining the targets in a duplex reaction did not affect quantification accuracy. Additionally, five cancer cell lines without FRS2 amplification, based on the CCLE database, were tested. The measured ratios of FRS2 to RPP30 copy numbers were approximately 1 (Fig. 2D), further supporting the assay’s reliability.

### Evaluation of assay quantitative linearity

**Fig. 3:**
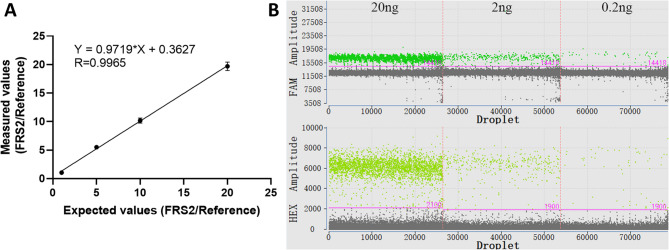
Performance of ddPCR Assay for FRS2 Copy Number Determination. **A** Linearity analysis of the ddPCR assay. The x-axis represents the expected copy number ratio, and the y-axis shows the measured values obtained from the assay; **B** Droplets plot of different template concentrations

Quantitative linearity was evaluated using mixtures of an artificial FRS2-containing plasmid and 5637 cell genomic DNA at predefined copy number ratios of FRS2 to RPP30 (1:1, 5:1, 10:1, 20:1). Theoretical values were plotted on the x-axis and measured copy number ratios on the y-axis (Fig. 3A). The results demonstrated strong linear correlation (R^2^=0.99), confirming excellent assay linearity across a wide dynamic range (Table 3).

**Table 3:** Copy number statistics of linear detection

Theoretical value	Repeat	Actual value		
FRS2:RPP30		FRS2 copy number (copies/ul)	RPP30 copy number (copies/ul)	FRS2:RPP30
1:1	1	132.79	121.95	1.09
	2	128.97	120.00	1.07
	3	117.26	111.50	1.05
5:1	1	691.80	124.09	5.57
	2	692.99	126.02	5.50
	3	658.95	120.59	5.46
10:1	1	1273.28	120.93	10.52
	2	1250.49	123.01	10.17
	3	1150.44	118.97	9.67
20:1	1	2431.12	128.37	18.94
	2	2062.70	101.17	20.39
	3	1985.21	100.54	19.74

### Minimum reliable input and precision of the ddPCR assay

The minimum reliable input DNA for the ddPCR assay was assessed using a series of serially diluted genomic DNA templates derived from healthy urine samples (Fig. 3B). The copy numbers of FRS2 and RPP30 were quantified (Table 4). At a total DNA input of 0.2 ng, the CV% reached 7.07% (> 5%). Therefore, 2 ng was defined as the minimum reliable input DNA for this assay. To further evaluate precision, FRS2/RPP30 ratios were measured using input amounts of 2 ng and 20 ng (Table 5). The intra-assay CV% was 2.58% and 3.75%, respectively, while the inter-assay CV% was 2.68% and 3.79%, demonstrating high reproducibility and precision of the assay across different input levels.

**Table 4:** Copy number statistics and CVs for serially diluted genomic DNA templates

Loading quantity	Repeat	FRS2 copy number (copies/ul)	RPP30 copy number (copies/ul)	FRS2:RPP30	CV%
20ng	1	224.35	203.93	1.10	4.61
	2	222.01	221.02	1.00	
	3	241.91	233.99	1.03	
2ng	1	25.15	23.78	1.06	3.42
	2	27.99	25.70	1.09	
	3	26.55	26.10	1.02	
0.2ng	1	9.46	7.51	1.26	7.07
	2	4.52	3.52	1.28	
	3	4.92	4.38	1.12	

**Table 5:** Precision evaluation of the detection system

	FRS2:RPP30					
	Loading quantity (20ng)			Loading quantity (2ng)		
Test day	repeat1	repeat2	repeat3	repeat1	repeat2	repeat3
Day1	1.10	1.00	1.03	1.05	1.01	1.08
Day2	0.99	1.04	1.04	1.01	0.98	1.01
Day3	1.03	1.02	1.05	0.95	1.06	1.03
Day4	0.99	1.03	1.02	1.00	1.04	1.03
Day5	1.06	1.03	1.06	0.99	1.11	1.02
Intra-assay CV%	2.58			3.75		
Inter-assay CV%	2.68			3.79		

### Comparison of ddPCR and FISH for FRS2 copy number detection

To define a normal reference range, urine sediment DNA from 18 healthy individuals was analyzed (Supplementary Table 1). The range was calculated as mean ± 3 SD (0.56–1.45), with ≥ 1.5 defined as amplification-positive. Seventeen formalin-fixed paraffin-embedded (FFPE) bladder cancer tissue samples were then analyzed using both ddPCR and FISH (Supplementary Fig. 1 and Supplementary Tables 2-3). FISH and ddPCR both detected FRS2 amplification in 6 cases (35.29%), while no amplification was observed in the remaining 11 cases (64.71%). The overall concordance between the two methods was 100% (Table 6), demonstrating a high level of agreement and supporting ddPCR as a reliable and efficient method for FRS2 copy number detection.

**Table 6:** Gold standard FISH to evaluate the FRS2 copy number detected by DdPCR

ddPCR	FISH			Specificity [%(95%CI)]	Sensitivity [%(95%CI)]	Kappa value
	Postive	Negative	Total			
Postive	6	0	6	100 (61–100)	100 (74–100)	1
Negative	0	11	11			
Total	6	11	17			

## Discussion

Copy number variations are prevalent across the human genome and have been implicated in tumorigenesis and cancer progression [31]. Amplification of the FRS2 gene has been reported in multiple malignancies, including bladder cancer and sarcomas, and is associated with poor clinical outcomes. Given its role in oncogenic FGF signaling, FRS2 has emerged as a potential therapeutic target [32]. Moreover, growing evidence supports its utility as a diagnostic and prognostic biomarker in cancer [10, 12–17]. However, no standardized assay has been established for the precise quantification of FRS2 amplification.

In this study, we established a ddPCR-based assay to quantify FRS2 copy number in bladder cancer specimens. The assay exhibited high reproducibility and precision. It is well documented that suboptimal binding between primers/probes and the DNA template can lead to the generation of intermediate droplets—commonly referred to as “rain”—which hampers accurate discrimination between positive and negative droplets and consequently affects copy number interpretation [31, 33]. In our assay, primers and probes were carefully designed to target exon 9 of FRS2, a conserved region, which minimized rain formation and enabled clear distinction between positive and negative droplet clusters, thereby ensuring accurate copy number determination (Fig. 2A).

FISH remains the current gold standard for assessing gene amplification in clinical settings. However, its application is constrained by high cost, technical complexity, semi-quantitative output, and reliance on expert interpretation [34]. These limitations underscore the need for a more accessible and objective method for quantifying FRS2 copy number.

In our validation using 17 FFPE bladder cancer samples, ddPCR showed excellent concordance with FISH results (100%, 17/17), demonstrating the reliability of our assay. Notably, in one sample, ddPCR detected a remarkably low FRS2:RPP30 ratio (0.05), which was inconsistent with the FISH-determined ratio of 1 (Supplementary Table 3). This discrepancy likely reflects partial deletion or DNA damage in the FRS2 gene region targeted by ddPCR, possibly due to tissue heterogeneity or fixation-induced degradation in FFPE samples. Since the ddPCR and FISH assays target different regions of the FRS2 locus, mismatched probe binding sites may also contribute to the discordance.

It is important to acknowledge that the relatively small sample size, especially in the FFPE validation cohort (*n* = 17), is a limitation of this study. The modest sample size may affect the statistical power and restrict the generalizability of our conclusions. Furthermore, it limited our ability to perform subgroup analyses, such as exploring associations between FRS2 amplification and clinicopathological parameters. Nonetheless, our findings provide preliminary but compelling evidence for the feasibility and reliability of ddPCR in copy number variation assessment. Further studies involving larger, independent cohorts are needed to validate our findings and to determine optimal interpretive thresholds for clinical application.

Compared with FISH, ddPCR offers several distinct advantages that enhance its potential for clinical application. It provides absolute quantification of target DNA without the need for standard curves. ddPCR is highly sensitive, capable of detecting low-abundance DNA, and provides precise results with minimal variability [35]. The workflow is simpler and faster than FISH, requiring less time and cost per sample, and is compatible with automation for high-throughput testing. Moreover, ddPCR generates objective digital data, minimizing interpretative subjectivity, and is more suitable for FFPE samples by circumventing common limitations of FISH, such as autofluorescence and poor probe penetration [36]. These advantages collectively support ddPCR as a robust and clinically applicable tool for accurate copy number analysis. Our findings provide the insight that ddPCR-based FRS2 quantification may facilitate the investigation of the association between FRS2 status and patient prognosis in clinical studies, and assist in predicting and identifying patients who are more likely to benefit from FRS2-targeted therapies.

## Supplementary Information

Supplementary Material 1: Supplementary Fig. 1. FISH analysis in 17 urothelial bladder carcinoma (UBC, the most common type of Bca) cases. The FRS2 probe was labeled with a red fluorophore and the CEP12 probe with a green fluorophore.

Supplementary Material 2: Supplementary Tables 1–3.

## Data Availability

Data is provided within the manuscript or supplementary information files.

## Abbreviations

ddPCR: Droplet digital PCR
FISH: Fluorescence in situ hybridization
Bca: Bladder cancer
NMIBC: Non-muscle-invasive
MIBC: Muscle-invasive
CNAs: Copy number alterations
FRS2: Fibroblast growth factor receptor substrate 2
TCGA: The Cancer Genome Atlas
CEP12: Centromeric region of chromosome 12
CV: Coefficient of variation
OS: Overall survival
DSS: Disease-specific survival
PFS: Progression-free survival
DFS: Disease-free survival
UBC: Urothelial bladder carcinoma
