# Supplementary figures and images for: Droplet digital PCR assay for precise determination of FRS2 gene copy number in bladder cancer
Source: BMC Cancer. 2025 Jul 24;25:1211. doi: 10.1186/s12885-025-14611-0 (PMC12291395; doi:10.1186/s12885-025-14611-0)

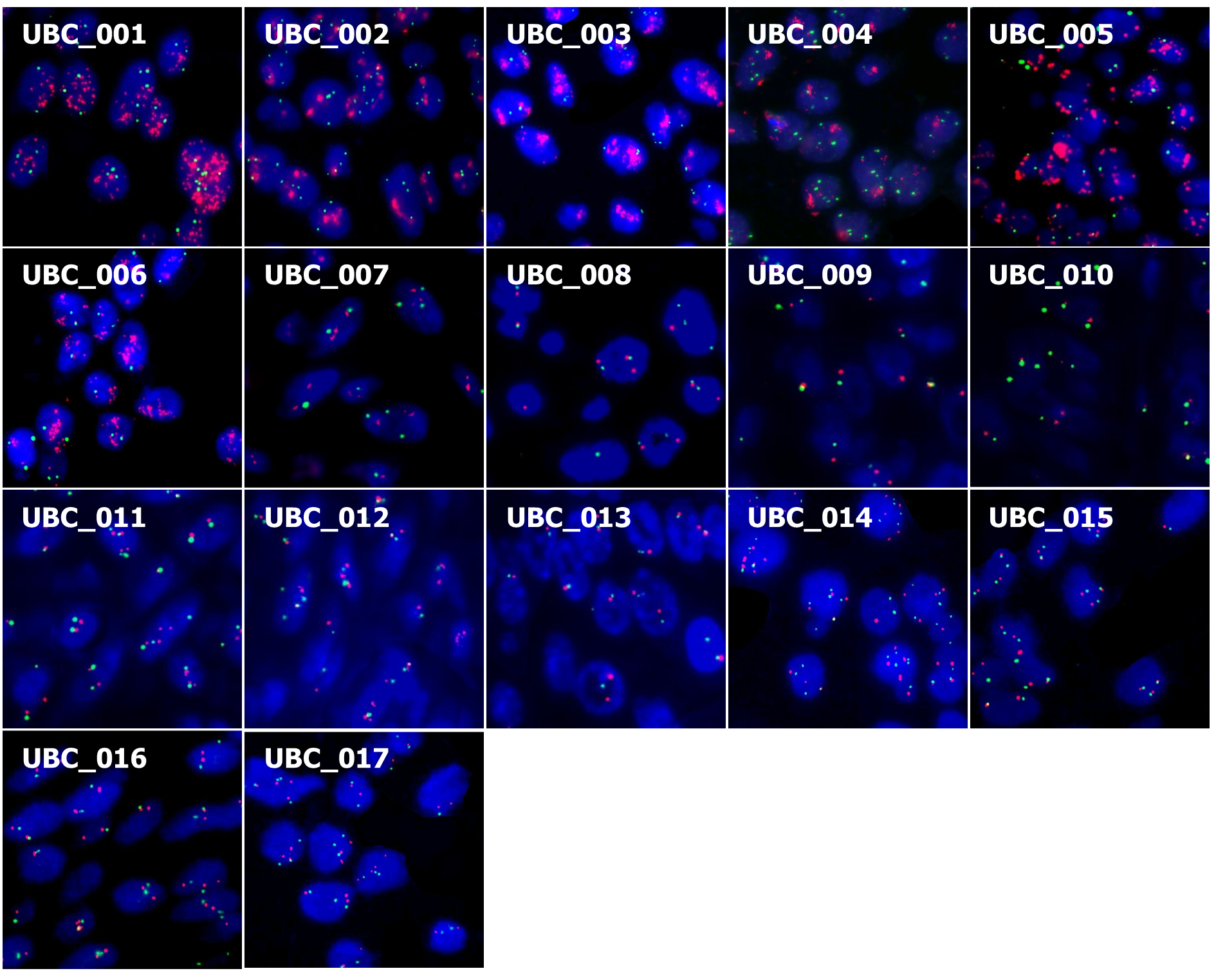

Supplement: Supplementary file 1 — Supplementary Material 1: Supplementary Fig. 1. FISH analysis in 17 urothelial bladder carcinoma (UBC, the most common type of Bca) cases. The FRS2 probe was labeled with a red fluorophore and the CEP12 probe with a green fluorophore. [file 12885_2025_14611_MOESM1_ESM.tif]
