# Supplementary material for: Droplet digital PCR assay for precise determination of FRS2 gene copy number in bladder cancer
Source: BMC Cancer. 2025 Jul 24;25:1211. doi: 10.1186/s12885-025-14611-0 (PMC12291395; doi:10.1186/s12885-025-14611-0)
Supplement: Supplementary file 2 — Supplementary Material 2: Supplementary Tables 1–3. [file 12885_2025_14611_MOESM2_ESM.docx]

**Supplementary table 1. Average copy number in urine sediment DNA from 18 healthy individuals based on two independent ddPCR experiments**

| Sample  No. | FRS2:RPP30 | Sample  No. | FRS2:RPP30 |
| --- | --- | --- | --- |
| 1 | 0.80 | 10 | 0.98 |
| 2 | 1.04 | 11 | 1.08 |
| 3 | 1.05 | 12 | 1.25 |
| 4 | 0.89 | 13 | 0.76 |
| 5 | 1.16 | 14 | 0.91 |
| 6 | 1.03 | 15 | 0.91 |
| 7 | 0.96 | 16 | 1.03 |
| 8 | 1.08 | 17 | 1.04 |
| 9 | 1.01 | 18 | 0.62 |

**Supplementary table 2. Clinical features of the 17 UBC cases**

| Patient id | Age range | TNM | NMIBC/MIBC | Primary/Relapsed |
| --- | --- | --- | --- | --- |
| UBC_001 | 60-69 | TaN0M0 | NMIBC | Primary |
| UBC_002 | 60-69 | T2N0M0 | MIBC | Primary |
| UBC_003 | 50-59 | T3N0M0 | MIBC | Primary |
| UBC_004 | 80-89 | T1N0M0 | NMIBC | Primary |
| UBC_005 | 70-79 | T1N0M0 | NMIBC | Primary |
| UBC_006 | 70-79 | T2N0M0 | MIBC | Primary |
| UBC_007 | 60-69 | T1N0M0 | NMIBC | Primary |
| UBC_008 | 60-69 | T4N2M0 | MIBC | Primary |
| UBC_009 | 60-69 | T4N0M0 | MIBC | Relapsed |
| UBC_010 | 60-69 | T1N0M0 | NMIBC | Primary |
| UBC_011 | 40-49 | T1N0M0 | NMIBC | Primary |
| UBC_012 | 70-79 | T1N0M0 | NMIBC | Primary |
| UBC_013 | 80-89 | T2N0M0 | MIBC | Primary |
| UBC_014 | 70-79 | T1N0M0 | NMIBC | Primary |
| UBC_015 | 70-79 | T2N0M0 | MIBC | Primary |
| UBC_016 | 70-79 | TaN0M0 | NMIBC | Primary |
| UBC_017 | 60-69 | T4N2M0 | MIBC | Primary |

**Supplementary table 3. Average copy number of 17 UBC cases based on two independent ddPCR experiments**

| Patient id | FRS2:RPP30 | Patient id | FRS2:RPP30 |
| --- | --- | --- | --- |
| UBC_001 | 7.50 | UBC_010 | 1.15 |
| UBC_002 | 2.51 | UBC_011 | 0.05 |
| UBC_003 | 4.53 | UBC_012 | 0.17 |
| UBC_004 | 7.09 | UBC_013 | 0.80 |
| UBC_005 | 4.32 | UBC_014 | 0.46 |
| UBC_006 | 2.31 | UBC_015 | 0.78 |
| UBC_007 | 1.19 | UBC_016 | 0.46 |
| UBC_008 | 0.34 | UBC_017 | 0.76 |
| UBC_009 | 0.24 |  |  |
